# Detecting DNA Depurination with Solid-State Nanopores

**DOI:** 10.1371/journal.pone.0101632

**Published:** 2014-07-02

**Authors:** Michael M. Marshall, Jan A. Ruzicka, Ethan W. Taylor, Adam R. Hall

**Affiliations:** 1 Joint School of Nanoscience and Nanoengineering, University of North Carolina at Greensboro, Greensboro, North Carolina, United States of America; 2 Department of Biomedical Engineering, Wake Forest University School of Medicine, Winston-Salem, North Carolina, United States of America; 3 Comprehensive Cancer Center, Wake Forest University School of Medicine, Winston-Salem, North Carolina, United States of America; Université d'Evry val d'Essonne, France

## Abstract

Among the different types of DNA damage that occur endogenously in the cell, depurination is especially prevalent. These lesions can initiate mutagenesis and have been implicated in a variety of diseases, including cancer. Here, we demonstrate a new approach for the detection of depurination at the single-molecule scale using solid-state nanopores. We induce depurination in short duplex DNA using acidic conditions and observe that the presence of apurinic sites results in significantly slower dynamics during electrokinetic translocation. This procedure may be valuable as a diagnostic for *in situ* quantification of DNA depurination.

## Introduction

Depurination is one of the most significant natural mechanisms of DNA degradation, occurring spontaneously under physiological conditions [1]. In this process, adenine and guanine bases are liberated when their N-glycosyl linkages to the deoxyribose backbone are hydrolyzed, resulting in an apurinic (AP) site. An estimated 2,000–10,000 purine nucleotides are lost per day in every human cell [2], most often as a result of thermal fluctuations, but potentially also through self-catalyzed mechanisms [3] or through the dissociation of DNA adducts [4]. While some AP sites may have functional roles in genetic recombination or nucleosome positioning [3], such lesions generally must be corrected through the base excision repair (BER) pathway [5], creating the potential for elevated mutation rates. As a result, AP sites have been linked to disease initiation, including cancers [4] and anemias [6]. A technique capable of linking relative DNA damage with various stages of disease could therefore be potentially transformative for diagnosis and treatment of disorders.

Most conventional methods for detecting AP sites rely on indirect measurement, such as screening for downstream mutations in bacteriophage [7] or gauging the ability of DNA to act as a template for PCR [8]. Recent efforts have also been made to detect by-products of depurination electrochemically [9]. More direct methods have been demonstrated as well, utilizing analytical techniques like high performance liquid chromatography [10], [11] or colorimetric assays [12]. However, these bulk assays are expensive and may mask small but important populations. A rapid technique with single-molecule sensitivity would be of significant value. Recently, An *et al*. demonstrated the detection of abasic sites using a protein channel [13]. This innovative approach has single-molecule sensitivity and can potentially be used to localize AP sites spatially within a known DNA sequence. However, some limitations exist with the technique, related to the chemical labeling method used, the reliance on a fragile lipid membrane, and importantly, the inability to investigate double-strand (ds) DNA.

**Figure 1:**
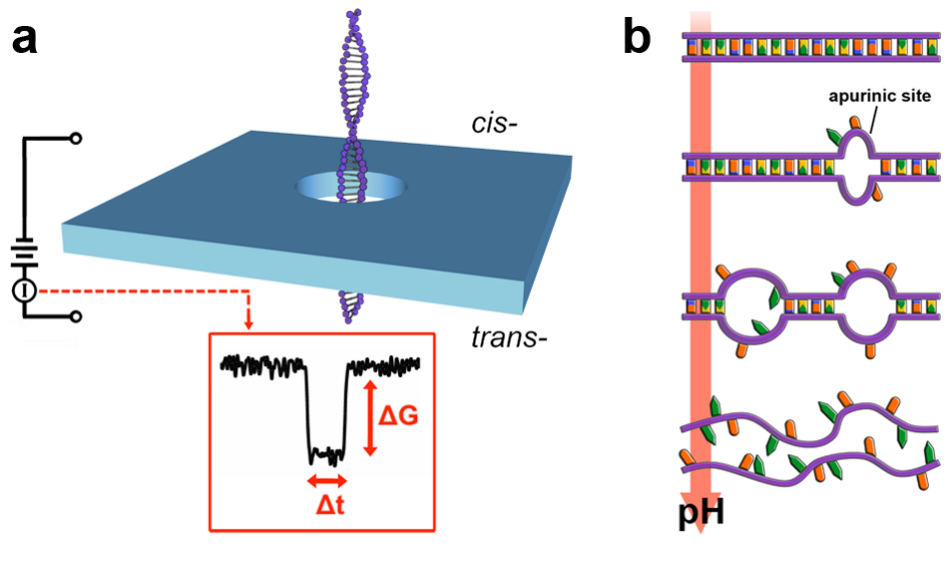
SS-nanopore detection of DNA depurination. (a) Schematic of the measurement system. A voltage applied across a membrane containing a single nanopore drives dsDNA from the cis- side to the trans- side. Inset: typical conductance blockade event shape with depth (*ΔG*) and duration (*Δt*) indicated. (b) Cartoon representation of pH-induced DNA depurination. Acidic conditions preferentially remove purine bases (G and A), which cause progressive loss of structure.

In this report, we demonstrate a new assay for the detection of depurination in short duplex DNA using solid-state (SS-) nanopores. SS-nanopores are an emerging technique [14], [15], [16] in which individual molecules are threaded electrokinetically through a narrow aperture fabricated in a thin, solid-state membrane. As they translocate, their characteristics can be determined through resistive pulse sensing (Fig. 1a). This approach has been used to measure a wide variety of biomolecules [17], [18], [19], biomolecular constructs [20], [21], and sub-molecular features [22], [23], and has recently been applied [24], [25] to epigenetic modifications, as well.

Here, we apply SS-nanopores to study unlabeled, heteropolymeric dsDNA and investigate the translocation dynamics of this model molecule with varying amounts of depurination induced through acid hydrolysis. We show that AP sites produce translocation durations that are up to an order of magnitude greater than what is measured for untreated material. The level of depurination can be coarsely estimated for each individual molecule as it translocates and bulk depurination levels can be assessed from a series of single-molecule measurements. As a result, our technique has potential as a sensitive, label-free diagnostic of AP site density in DNA.

Experimental methods to induce depurination in DNA have become well-established, given its fundamental significance. Both heat and chemical treatments (e.g. ethyl ethane sulfonate) are effective depurinating agents that remove guanine preferentially from DNA [7], [26]. However, solvent chemistry and temperature may affect SS-nanopore translocations independently, and so we instead vary the density of AP sites using pH (Fig. 1b). Acidic conditions are known to protonate DNA and hydrolyze purine residues, so pH is used commonly to induce depurination in biochemical assays like Southern blotting [27]. Therefore, through measurements across a broad pH, we are able to investigate the effects of substantial differences in the amount of depurination.

In order to probe these effects systematically, we use a series of SS-nanopores (four separate devices) ranging in diameter from 5–6 nm to translocate 61 bp DNA in high-ionic strength measurement solution (1 M KCl) over a pH range from 2 to 10. In each case, the duplex DNA is incubated at a given pH for 1 hr before being introduced to the grounded *cis* side of a pore (Fig. 1a). The application of a positive voltage (400 mV) to the *trans* side is then used to induce translocations. Threading of molecules through the SS-nanopore is manifested by brief, transient blockade events (Fig. 1a, inset) in the measured trans-pore ionic conductance that are described by a characteristic depth (*ΔG*) and duration (*Δt*). We record a constant succession of blockade events under all investigated conditions.

## Materials and Methods

Commercial silicon chips, each supporting a free-standing SiN membrane, were purchased from Norcada (Edmonton, Canada) and used as delivered for nanopore fabrication. Membrane thickness was measured to be 24.5 nm using ellipsometry. A single nanopore with a diameter of 5–6 nm was produced in a membrane using a Helium ion microscope (Carl Zeiss Orion PLUS, Peabody, MA). Pore formation was carried out as reported previously [28].

Duplex 61-mer DNA with a sequence of 5′-AGTACTGCTAGCAATGCCCTGGAACGGAATTCTTAATAAAGATGTATCATTCTGCAGTACT-3′ was purchased from Integrated DNA Technologies (Coralville, IA), re-suspended at a concentration of 4 mg/ml in 10 mM Tris buffer, 1 mM EDTA (pH 8), and stored at −20°C. Each pH treatment was prepared by adding (at a 1∶100 ratio) this stock DNA to 1 M KCl measurement solution at the desired pH and incubating the mixture at room temperature for 1 hour. Solution pH was adjusted by adding sodium carbonate/bicarbonate (pH 10), Tris (pH 8), sodium acetate (pH 6), sodium citrate (pH 4), or HCl (pH 2).

To prepare the SS-nanopore device for translocation measurements, a chip containing a single SS-nanopore was rinsed with acetone and ethanol, dried under a nitrogen stream, and treated with oxygen plasma (150 W) for 3 min on each side. Immediately after plasma treatment, the chip was seated inside a custom Ultem 1000 flow cell and wetted by adding the same 1 M KCl electrolyte used to prepare the DNA, except for the additional pH 4 treatment noted in the text. These were placed in high humidity to reduce evaporation and left at room temperature to equilibrate for the same one-hour DNA incubation period. For the additional pH 4 treatment, the pore was equilibrated in 1 M KCl at pH 8.

DNA translocations were performed by introducing equilibrated DNA solution (at a concentration of ∼40 ng/µl) into the *cis* flow cell reservoir and applying +400 mV to the *trans* chamber using a patch-clamp amplifier (Axopatch 200B, Molecular Devices, Sunnyvale, CA) with a four-pole Bessel filter of 100 kHz. The electrical signal was sampled at 250 kHz and subjected to an additional low-pass filter of 50 kHz prior to analysis using custom LabView software. The total numbers of events considered were as follows: *n* = 714 (pH 10), 662 (pH 8), 552 (pH 6), 423 (pH 4) and 1852 (pH 2).

The gel electrophoresis assay was performed using equal amounts of 61 bp dsDNA in each lane. For this measurement, dsDNA was incubated for 1 hr at a given pH as described above and then loaded directly onto a 1% agarose gel prepared with a Tris/Borate/EDTA buffer solution (pH 8.3) and an intercalating dye (Ethidium Bromide Solution, Promega Biosciences, San Luis Obispo, CA).

## Results and Discussion

**Figure 2:**
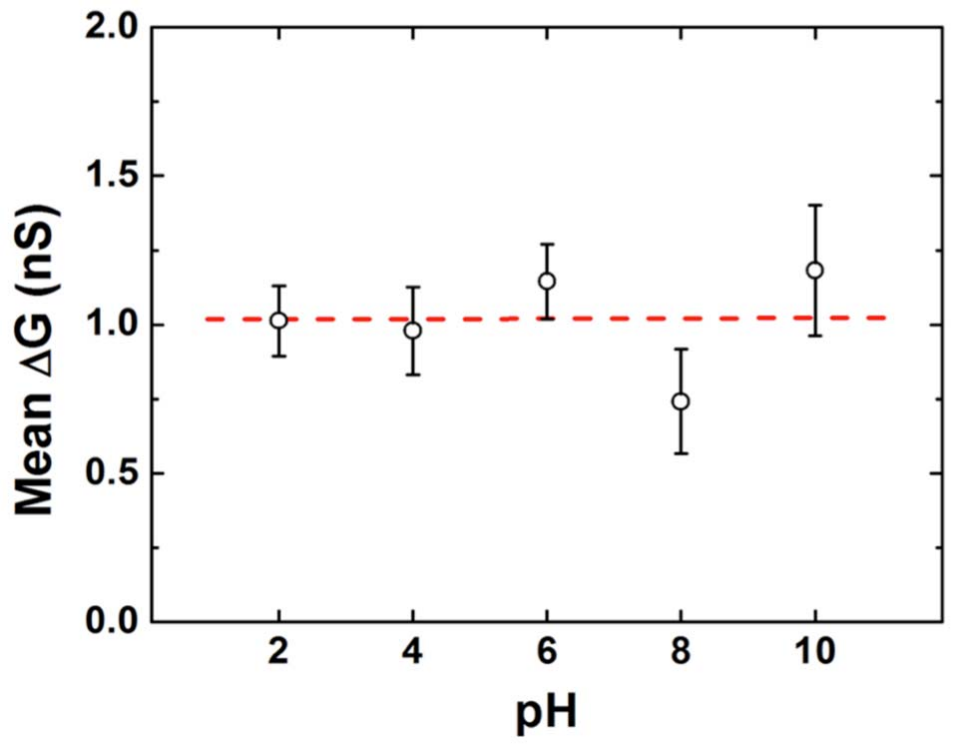
Conductance blockade depth across the pH range. Mean conductance change measured for 61-10. No significant variation is observed. Error bars represent the width of a Gaussian fit to the data and the dashed line represents the average value from all data sets.

**Figure 3:**
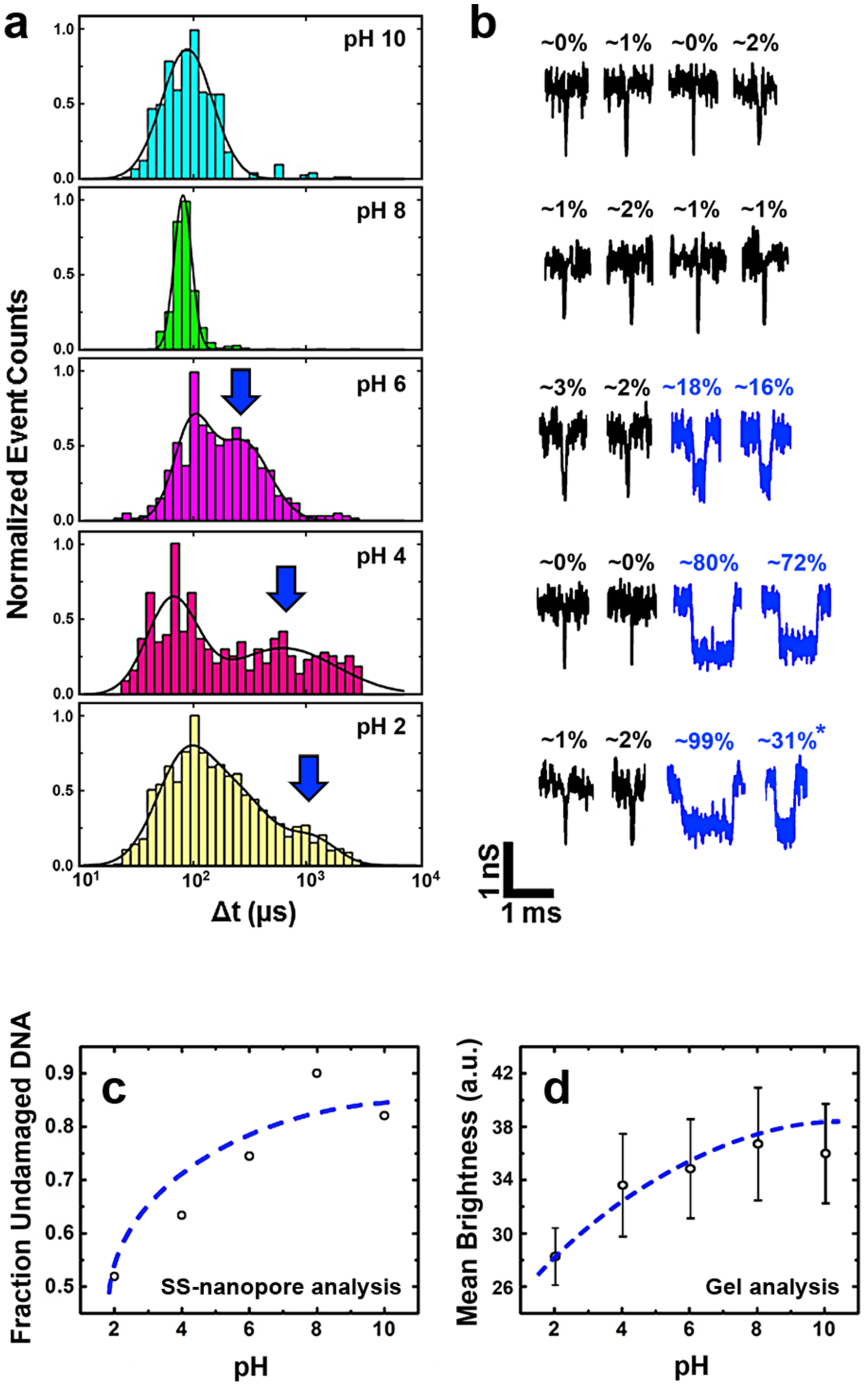
Event durations across the pH range. (a) Event duration histograms for 61 bp DNA translocation events from pH 10 (top) to pH 2 (bottom). Total numbers of events considered are n = 714 (pH 10), 662 (pH 8), 552 (pH 6), 423 (pH 4) and 1852 (pH 2). The black lines represent Gaussian fits to the data (pH 10 & 8: single peak; pH 6 & 4: two peaks; pH 2: three peaks). (b) Example traces of typical events measured at the adjacent pH level. Black traces represent undamaged (low *Δt*) events. For pH 6, 4 and 2, the blue traces are typical events derived from the long duration (i.e. depurinated) population, indicated by a blue arrow on the histograms. For each event trace shown, the label indicates a coarse approximation of the relative amount of depurination. Note that the final event at pH 2 (marked with *) may be indicative of DNA fragmentation (see text). (c) Fraction of translocation events in the undamaged population and (d) intensity of the DNA band on a gel (see also Supplementary Information), each measured over the entire range of pH investigated. Dashed lines are logarithmic fits to the data.

As pH is reduced, we find only negligible changes in the depth of measured events, yielding a mean *ΔG* of 1 nS (Fig. 2). This value agrees with numerous other reports of dsDNA translocations under comparable high-ionic strength conditions [17], [19], [29] and is consistent with simple size-exclusion [30]. Importantly, however, we find that translocation duration changes considerably over the same pH range. Under all measured levels of pH (Fig. 3a & b), we observe a significant population of events with a mean *Δt* of ∼70-100 µs. We attribute this consistent duration to the passage of native, non-degraded dsDNA, which should be present to some degree under all conditions.

However, at low pH (≤6), an additional population emerges with much longer duration. We note that a small number of slow events (∼200 µs) is also observed at pH 8, but these events are rare and thus cannot be adequately fitted with a Gaussian distribution. At pH 6, we find a population with a mean *Δt* of 280 µs; at pH 4, a mean *Δt* of 610 µs; and at pH 2, a mean *Δt* of 1140 µs, an order of magnitude greater than the unmodified duration. We suggest that the longer translocation times occur because the native DNA helix is disrupted as purines are liberated at low pH, resulting in unstructured regions characterized by missing base-pairs and strand separation. Both of these consequences may contribute to greater interaction with the pore walls and thus slower translocation times. First, unpaired nucleotides opposite abasic sites can rotate more freely and interact directly with the pore. Second, separated strands occupy a larger effective volume, potentially increasing the amount of time the DNA is in contact with the pore. Under extreme conditions of pH, the exposed phosphodiester backbone and increased inter-molecular coupling can facilitate complex configurations that are often unable to pass through the pore. Indeed, for many devices, we observe irreversible clogging of the pore at pH 2 (data not shown), which we attribute to attempted threading of DNA with extensive structural damage.

We note that the data taken at pH 2 yields a large number of events with durations intermediate to the two dominant populations (see Fig. 3a, bottom). We attribute these to a complete loss of duplex structure into single-strand DNA polymers and possible fragmentation, which is known to occur at higher rates under acidic conditions [31]. Translocation of these smaller molecules will have the effect of reducing the measured *Δt* from the depurinated level. We also note that there is some variation in the distribution widths of the fast translocation (70-100 µs) population between various pH levels, with the narrowest distribution occurring at pH 8. We suggest that this is because the dsDNA is most structurally stable under this near-physiological condition; at pH 10, for example, electrostatic repulsion of the backbones can cause localized denaturation and thereby produce variation in event dwell time.

We find that a decreasing proportion of recorded events falls inside the limits of the undamaged population as solvent conditions are made more acidic (Fig. 3c). This is in accordance with expectations, considering that the level of DNA depurination is known to increase rapidly as pH is reduced [2]. We also investigate the same molecules treated at each pH by gel electrophoresis (see Fig. S1). Since dye intercalation will be hindered by loss of helical structure, we anticipate that the amount of DNA that can be visualized on such a gel will be reduced as depurination density increases. Analysis of band intensity confirms this (Fig. 3d), yielding a qualitatively similar logarithmic dependence on pH. This is a reasonable relation considering that pH is a logarithmic measure of solution hydronium ion content. This result provides secondary confirmation of our approach.

Importantly, due to the nature of SS-nanopore measurements, *Δt* can be assessed for individual events, providing a rough estimate of the relative density of AP sites present in each molecule. Fig. 3b shows example traces of dsDNA events with low (black) and high (blue, where applicable) *Δt* for each pH level investigated. If we assume that the minimum duration (∼80 µs) represents undamaged dsDNA and that the maximum duration observed (∼1 ms) represents DNA that is almost completely depurinated, then the relative level of depurination for all intermediate events can be approximated by event *Δt* (see Fig. 3b). While we stress that this estimation is coarse, we find mean depurination levels of ∼21% at pH 6 and ∼58% at pH 4.

A possible alternative explanation for the observed differences in event duration could be changes in the net electrical forces at play in the SS-nanopore caused by protonation. Firnkes *et al*. [32] demonstrated that the electrophoretic and electroosmotic forces acting on proteins could be modified or even reversed due to pH-induced surface charge effects in a comparable system. However, these measurements were performed in low ionic strength solution. The extent to which pH can alter translocation dynamics depends, in part, on the relative zeta potentials of the nanopore and analyte. Since Manning condensation [33] reaches saturation in high ionic strength solvents, charged surfaces are better shielded and zeta potential changes are inhibited under these conditions. As a result, electrophoretic forces are expected to remain relatively constant over a wide range of pH and electroosmotic flow inside the nanopore is suppressed. Indeed, recent work by Anderson, *et al*. [34] showed that the translocation dynamics of dsDNA in 1 M KCl were insensitive to pH unless the SS-nanopore was functionalized with an organic coating.

In order to isolate effects of dsDNA structural changes (i.e. depurination) from these purely electrical effects, we perform an additional experiment in which we first incubate 61 bp DNA in 1 M KCl at pH 4 as above, but subsequently adjust the solution to 1 M KCl, pH 8 prior to SS-nanopore measurement. In this way, the irreversible structural modifications can be investigated under the same translocation conditions that yield a single, well-defined *Δt* population when untreated dsDNA is measured (see Fig. 3a). This allows any pH-induced counterion effects (such as electroosmosis) to be separated from the depurination process itself.

**Figure 4:**
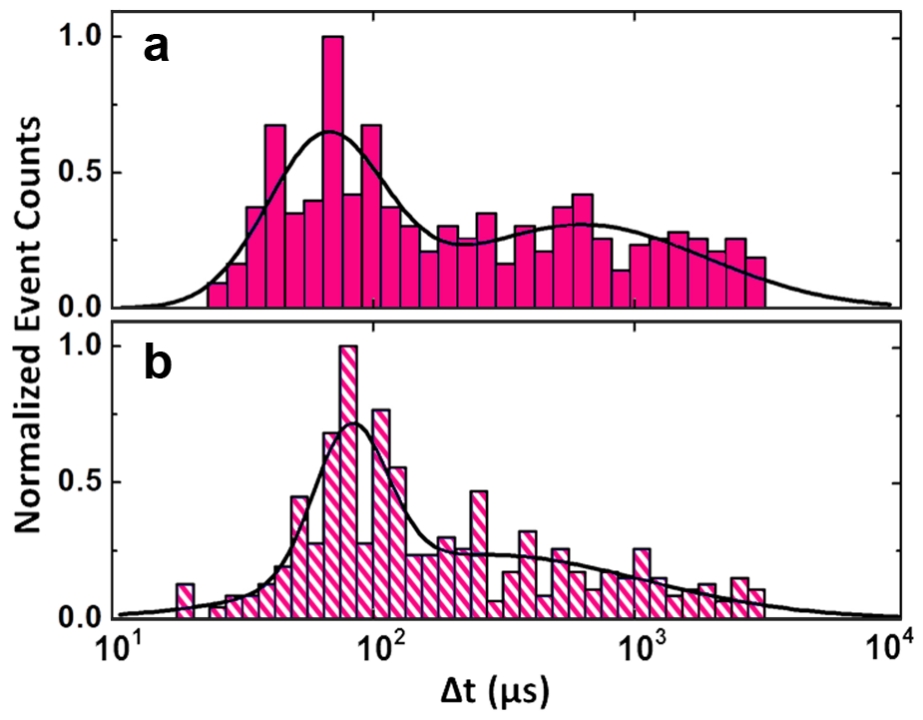
Isolating structural factors from solvent effects. Dwell time histograms for two different SS-nanopore measurements of dsDNA incubated at pH 4. (a) Incubation and measurement in 1 M KCl at pH 4 (same data as pH 4 histogram in Fig. 3) and (b) incubation in 1 M KCl at pH 4 and measurement in 1 M KCl at pH 8 (n = 408).

As shown in Fig. 4, these measurements yield a *Δt* histogram similar to that of translocations performed using pH 4 measurement solution, with a significant peak around 68–95 µs and a large population of events extending to longer durations. The locations of the long *Δt* population under both conditions are within error of each other. These translocation results support our hypothesis that the increased *Δt* we observe using a SS-nanopore analysis of low-pH events is caused by depurination-induced changes in the DNA structure.

## Conclusions

In conclusion, we have demonstrated that SS-nanopores can be used to characterize depurination in short duplex DNA molecules. We used low pH conditions to hydrolyze glycosidic bonds in 61 bp DNA, releasing purine nucleotides in the process. We found that this degradation produced significant increases in the duration of conductance blockades, showing that depurinated DNA translocates up to an order of magnitude more slowly than undamaged molecules, on average. This observation was attributed to a progressive loss of the double-stranded helix, which intensifies confinement effects due to open regions of single-stranded structure where unpaired nucleotides can come into direct contact with the SS-nanopore. This facilitates stronger interactions between threading molecules and the pore, inhibiting translocation speeds. Our approach is fast, label-free, and can be used for a coarse determination of either the overall level of depurination within a collection of dsDNA or the degradation of individual translocating molecules. While assumptions are currently required for this type of characterization, further study of the system will enable direct quantification of abasic sites. Given that depurination is a continual process, this detection technique could have useful applications in a wide variety of fields that rely on DNA analyses, including forensics. Finally, since AP sites can lead to the initiation of diseases, such as cancer, SS-nanopore detection of depurination may have future clinical relevance as a diagnostic tool.

### Supplementary Data

Image of gel analyzed in Fig. 3d.

## Supporting Information

Figure S1 **Gel analysis of DNA across a pH range.** Gel electrophoresis performed on 61 bp DNA subjected to various pH conditions, indicated at the top of each lane. The blue arrow indicates the position of 61 bp DNA.
(TIF)
