# Supplementary figures and images for: Detecting DNA Depurination with Solid-State Nanopores
Source: PLoS One. 2014 Jul 2;9(7):e101632. doi: 10.1371/journal.pone.0101632 (PMC4079296; doi:10.1371/journal.pone.0101632)

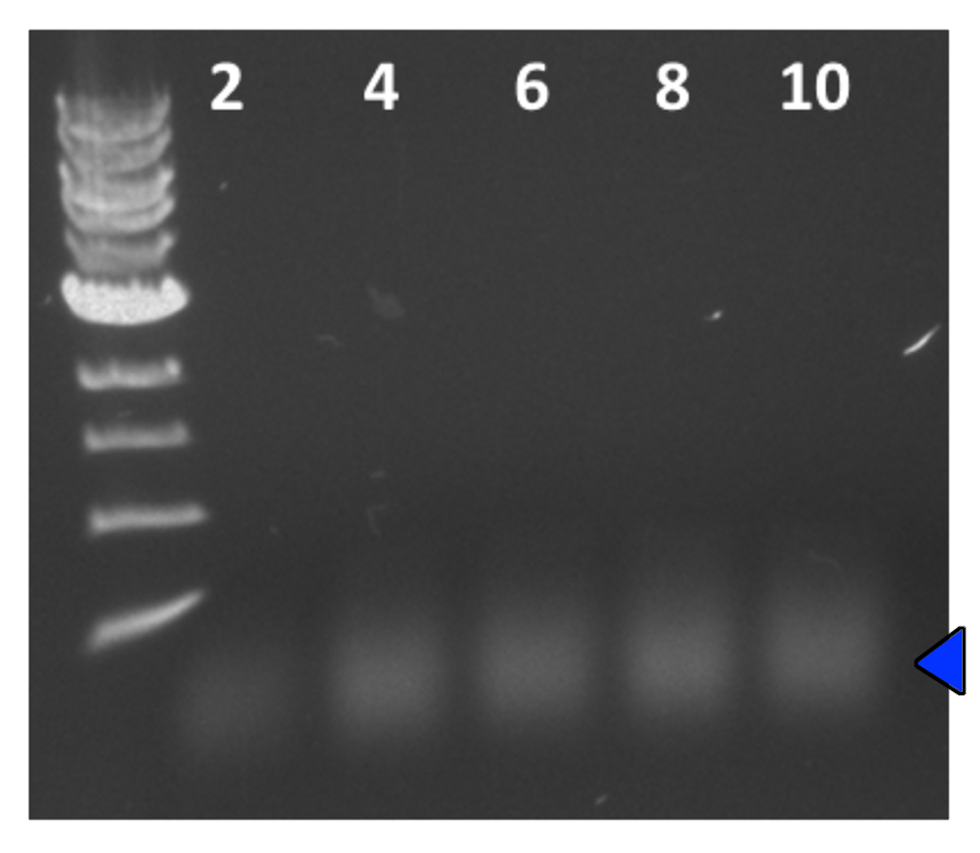

Supplement: Figure S1 — Gel analysis of DNA across a pH range. Gel electrophoresis performed on 61 bp DNA subjected to various pH conditions, indicated at the top of each lane. The blue arrow indicates the position of 61 bp DNA. (TIF) [file pone.0101632.s001.tif]
